# The lived experience of stigma and parkinson’s disease in Kenya: a public health challenge

**DOI:** 10.1186/s12889-023-15278-7

**Published:** 2023-02-20

**Authors:** Natasha Fothergill-Misbah

**Affiliations:** Faculty of Social Sciences, University of Southampton, Southampton, UK

**Keywords:** Parkinson’s disease, Stigma, Witchcraft, Discrimination, Structural violence, Awareness, Supernatural, Stereotypes

## Abstract

**Background:**

As a disease characterised by non-motor and very visible motor symptoms, Parkinson’s disease has been associated with multiple forms of stigma, while awareness about the disease globally remains low. The experience of stigma relating to Parkinson’s disease from high-income nations is well-documented, while less is known about low- and middle-income countries (LMICs). Literature on stigma and disease from Africa and the Global South has described the added complexities people face resulting from structural violence, as well as perceptions about symptoms and disease associated with supernatural beliefs, which can have significant implications for access to healthcare and support. Stigma is a recognised barrier to health-seeking behaviour and a social determinant of population health.

**Methods:**

This study draws on qualitative data collected as part of a wider ethnographic study to explore the lived experience of Parkinson’s disease in Kenya. Participants include 55 people diagnosed with Parkinson’s and 23 caregivers. The paper draws on the Health Stigma and Discrimination Framework as a tool to understand stigma as a process.

**Results:**

Data from interviews identified the drivers and facilitators of stigma, including poor awareness of Parkinson’s, lack of clinical capacity, supernatural beliefs, stereotypes, fear of contagion and blame. Participants reported their lived realities of stigma, and experiences of stigma practices, which had significant negative health and social outcomes, including social isolation and difficulty accessing treatment. Ultimately, stigma had a negative and corrosive effect on the health and wellbeing of patients.

**Conclusion:**

This paper highlights the interplay of structural constraints and the negative consequences of stigma experienced by people living with Parkinson’s in Kenya. The deep understanding of stigma made possible through this ethnographic research leads us to see stigma as a process, something that is embodied and enacted. Targeted and nuanced ways of tackling stigma are suggested, including educational and awareness campaigns, training, and the development of support groups. Importantly, the paper shows that awareness of, and advocacy for the recognition of, Parkinson’s globally needs to improve. This recommendation is in line with the World Health Organization’s Technical Brief on Parkinson disease, which responds to the growing public health challenge posed by Parkinson’s.

## Introduction

Parkinson’s disease^1^ is a neurodegenerative condition characterised by motor symptoms (slow movement, tremor, rigidity and imbalance) and a spectrum of non-motor complications (including neuropsychiatric symptoms, autonomic dysfunction and behavioural disorders) [1]. Although the motor symptoms of Parkinson’s can be barely noticeable at onset, the disease is notably characterised by very *visible* symptoms that progress over time, impacting functioning and resulting in significant disability [2] that can be accompanied by social and self-stigma [3].

The prevalence of Parkinson’s globally has doubled in the last 25 years and is expected to affect 12.9 million people by 2040 [4], posing a growing public health challenge [5]. However, true global numbers are difficult to estimate [6] owing to low rates of diagnosis [7] and limited epidemiological evidence [8]. As a condition associated with ageing, global improvements in life expectancy [9] are contributing to the disease’s label as ‘the fastest growing neurological disorder in the world’[10].

Although existing data would suggest that Parkinson’s is not as prevalent in regions such as sub-Saharan Africa – for example, age-standardised prevalence estimates in ‘high-income’ North America are five times that of sub-Saharan Africa [6, 11] – prevalence is almost certainly rising. The challenges currently facing individuals in Africa are poor awareness, lack of access to biomedical treatment [12] and the social stigma people with Parkinson’s and their families experience [13–15]. Therefore, understanding how Parkinson’s is negotiated in daily life in low- and middle-income country settings is crucial in order to ensure positive health outcomes.

### Stigma definition and frameworks

Stigma associated with Parkinson’s has been observed and reported across the world (see review by Maffoni et al. [16]). Goffman has defined stigma as the identification of people based on particular traits (physical, behavioural or social) that are perceived as different to “normal” groups, and the subsequent “disqualification” of individuals [17]. It is a complex and powerful phenomenon that can arise from cultural and community perceptions and beliefs about disease [18] which are often grounded in social inequalities [19]. Stigma has been linked to evolutionary pressures to distinguish between beneficial and detrimental social connections and avoid those who may carry communicable pathogens, for example [20]. Stigma is a well-documented barrier to health-seeking behaviour [21] and can negatively affect psychological and physical well-being, social status, and exacerbate poor health [17].

Stigma is a *“constantly changing social process”* (p.14) [22], and understanding stigma requires consideration of the social, political, historical and economic roots of stigma and associated discrimination and poor health [23]. Stangl et al. recently developed a ‘Health Stigma and Discrimination Framework’ to explore stigma *across* diseases – striving to move away from the focus of health-related stigma frameworks on individual health conditions [21]. The framework encompasses stigmatisation across the socioecological spectrum, involving drivers and facilitators, stigma ‘marking’, manifestations (such as discrimination), outcomes and wider health and social impacts. The framework highlights commonalities across conditions, and identifies areas for research, intervention and policy.

### Stigma and structural violence

Considering stigma within a broader social and political understanding involves a focus on the processes of social and health inequality, social exclusion and social injustice – acts of “violence” [24]. The concept of “structural violence” [25] has been defined as *“social arrangements that put individuals and populations in harm’s way. The arrangements are structural because they are embedded in the political and economic organization of our social world; they are violent because they cause injury to people”* (p.1686) [26]. Using structural violence as a framework to understand stigma considers the unequal distribution of power in society that disadvantages individuals [24], focussing on stigma as a “fundamental cause” of disease or poor health [23, 27, 28].

Structural violence has been used to understand social inequalities in which stigma is embedded, with a particular focus on HIV and AIDS [19]. Examples include understanding the mechanisms that place individuals at risk of HIV acquisition or on factors affecting disease course [26], which is where the application of structural violence to Parkinson’s is useful. Although forms and consequences of structural violence exist with Parkinson’s, such as inequalities in exposure to risk factors (for example, pesticides and industrial solvents [29, 30]), the focus of this article is on the drivers, facilitators and manifestations of stigma, and resulting disparities in access to therapy, health services and medication, exclusion from society, and potential negative health outcomes.

### Stigma and parkinson’s

Stigma can involve “enacted” stigma, discrimination by others towards someone because of their condition, for example, because of visible symptoms, and “felt” or “self” (“internalised”) stigma, associated with ‘shame’ or ‘embarrassment’ [31]. The visible or physical manifestations of Parkinson’s – tremor, slowed speech or dyskinesias – can “discredit” and “devalue” individuals [17], marking them as “different”, resulting in alarm, avoidance and labelling of individuals by others (enacted stigma) [3]. For example, in Israel, Posen et al. reported how women with Parkinson’s described their bodies as “traitors”, revealing their condition to the public [32], while in the USA, people with Parkinson’s have been labelled as “drunk” [33]. Stereotypes about Parkinson’s have also been described as drivers of enacted stigma [16]. For example, in Tanzania, Mshana et al. found that because Parkinson’s was associated with ‘old age’, two younger individuals in their study – aged 41 and 57 – experienced greater stigmatising perceptions [13], similar to findings in the USA [33].

In Iran, body image has been reported to result in embarrassment and social isolation – felt or self-stigma – among individuals with Parkinson’s [34]; similar to descriptions in The Netherlands of Parkinson’s being “a problem of shame” (p.196) due to individuals’ self-perceived physical dependency [35]. Feelings of self-stigma can exacerbate the impact of enacted stigma, disrupting autonomy and social connectedness or engagement, and affecting social interaction [3]. Stigma has also been described in relation to a changing self [36] and loss of social roles, for example, the perception of no longer being able to provide for the family [37]. Secondary or ‘associative’ stigma [21] among caregivers has also been reported in Parkinson’s – in Tanzania, Mshana et al. described how an entire family was stigmatised because of the individual’s condition [13].

Several scales have been developed to measure stigma in Parkinson’s, which can offer useful insights into the intensity, and core experiences, of stigma. These include components of the Parkinson’s Disease Questionnaire (PDQ-39) and Parkinson’s Disease Quality of Life Questionnaire (PDQL) [38]. For example, in China, Lin et al. investigated the evolution of self-stigma in early-stage Parkinson’s (using PDQ-39), identifying a decrease in self-stigma with increasing disease progression [39]. The Stigma Impact Scale and Stigma Experience Scale have also been adapted to identify and measure perceived stigma in Parkinson’s (see Burgener and Berger [40]). Due to the subjective nature of stigma, measurements can be difficult, and scales may not fully capture the experience of stigma across cultures. The cross-cultural validation of these tools, as has been done for the PDQ-39 in Egypt [41], for example, would assist further with further cross-context comparisons.

In addition to the evidence presented here – most of which comes from higher-income countries [16] – findings from sub-Saharan Africa relating to Parkinson’s have identified additional stigmatising perceptions associated with beliefs about the origins, or causes of, visible symptoms, namely “witchcraft” and “curses” [13–15]. For example, in central Uganda, urban and rural adult participants believed that Parkinson’s was contagious or a form of “insanity” that could be caused by *“touching the mother-in-law”* (p. 6) [14]. In South Africa, similar perceptions about Parkinson’s associated with witchcraft were seen [15]. Subsequently, 45% of the 98 members of the public in the study sample believed that people with Parkinson’s with dyskinesia should not live in the community. However, despite the profound repercussions of stigma, we know very little about the lived experience of stigma and Parkinson’s, the consequences of discrimination, and the wider health and social impacts of exclusion; particularly in places like sub-Saharan Africa (and the wider Global South).

The focus of this paper is to explore stigma relating to Parkinson’s in Kenya across the socioecological spectrum [21], using structural violence as an explanatory concept. Key research questions are:

1. What drives, or facilitates, stigmatising perceptions related to Parkinson’s?
2. How is stigma experienced by people with Parkinson’s?
3. What are the outcomes and impacts of stigma for people with Parkinson’s and their families?

The paper addresses each of these questions and ends with reflections on how to address stigma related to Parkinson’s. To the best of my knowledge, this paper is the first to describe in detail the perceptions, experiences, and consequences of stigma for people with Parkinson’s in any low- and middle-income country context.

## Methods

Data presented come from a larger ethnographic study of the lived experience of Parkinson’s disease. Ethnography, as a methodology, can be used to acquire knowledge to study socio-cultural contexts, processes and meanings [42], with the aim of generating a “thick description” and holistic understanding of people’s lives.

Fieldwork was carried out over ten months in Kenya (2018–2019) and involved participant observation and in-depth semi-structured interviews with people with Parkinson’s (n = 55), family members/caregivers (n = 23), healthcare professionals (n = 22) and herbal healers (n = 3). Most of the fieldwork period was spent in Nairobi and Mombasa, with shorter periods in rural central Kenya and western Kenya. This paper specifically addresses the experiences of stigma by people with Parkinson’s and their families. Therefore, the methods described here relate to the interviews with these groups, from which the data were derived.

### Participants

Participants (see Table 1 for profile) were identified and invited to the study by the author through two pathways: (1) an existing Parkinson’s support group in Nairobi, and (2) neurology clinics in Nairobi (one private, one public) and in Mombasa (one private). At the clinics, the author (with permission and assistance of staff) identified people with a confirmed diagnosis of Parkinson’s through clinic files and contacted them with an invitation to participate. At the support group, the author was offered the opportunity to discuss the research and attendees (people with Parkinson’s and caregivers) were invited to approach the author if they were interested in participating. Caregivers were also invited to participate if the person with Parkinson’s (as the primary participant) consented to this.

**Table 1:** Profile of study participants

Participants	Urban/Rural split	Male/Female split
People with Parkinson’s (n = 55)	39/16	32/23
Caregiver (n = 23)	19/4	7/16

People with Parkinson’s ages ranged from 33 to 81-years-old (median age 66.5) – ten participants were younger than 60-years-old at the time of the study. All had travelled (up to 200 km) to Nairobi or Mombasa to attend consultations or support group meetings – participants were limited to individuals with sufficient resources and connections to travel to the city and access clinics. This was a necessary but unavoidable limitation of the study, as the majority of rural Kenyans with Parkinson’s, and those with lower social and financial capital, remain undiagnosed and therefore ‘invisible’. Without a door-to-door community study, it is difficult to recruit individuals with Parkinson’s who do not access formal services.

Participants were not asked to disclose their household income, however, people with Parkinson’s in the sample had very different financial and social resources and living situations (i.e., those attending private vs. public clinics). Five lived alone and most (n = 15) lived with one other person, reflecting the urban, nuclear participants recruited. Twenty-seven identified a main family caregiver. Most were previously employed in the formal or public sector (e.g., teacher) and 13 were self-employed (e.g., owned a small fruit and vegetable stall).

### Data collection

All participants were invited to take part in a semi-structured interview with the author. Initial interviews with people with Parkinson’s (n = 55) were not audio recorded to avoid the risk of losing rapport, and detailed hand-written notes were taken instead. Nine were carried out in Kiswahili and required an interpreter (one in Nairobi and 8 in Mombasa) to ensure accurate translations and limit any loss of meaning in the process. All interviews with caregivers (n = 23) and follow-up interviews with people with Parkinson’s (n = 9) were audio-recorded, and all but one were conducted in English. Follow-up interviews took place when there was scope for further exploration after preliminary analysis of interviews, or when a participant’s circumstances had changed over the course of fieldwork, e.g., change of living situation or worsening of condition.

Interviews took place at a convenient location for the participants, including clinics, cafes, and participant homes. Written, informed consent was obtained after participants read the information sheet, or had the sheet read to them. All interviews took a biographical approach [43], exploring participants’ experiences of Parkinson’s, using probes as conversation guides where necessary to assist recollection. Caregiver interviews took place without the individual with Parkinson’s present, which allowed them to discuss their experiences freely.

### Analysis

Qualitative data were analysed using inductive thematic analysis [44], aligning with the interpretative nature of ethnography, and allowing themes to be identified from the data while maintaining the depth and originality of individual stories and experiences. Analysis was iterative and entailed constant reviewing of transcripts and personal reflections while in the field. This systematic, ‘bottom up’, reflexive approach considered the political-economic, sociocultural, historical and cultural context of the study setting. Following Braun and Clarke’s six phases of thematic analysis, recordings were listened to and transcribed by the author, facilitating immersion in the data. Hard copies of transcripts were coded line by line, by hand, and then conceptualised empirical and theoretical codes were collated into main and sub-themes after iterative reviewing and refining. The final detailed analysis tells an interpretative story of the data, with quotations used as representations of wider responses. Continuous reflexivity throughout the research process allowed for reflection on ideas and experiences [45].

## Results

Using The Health Stigma and Discrimination Framework [21], the results discuss three main themes: first, the reported drivers and facilitators of stigma, including lack of awareness, blame, supernatural beliefs and stereotypes; second, participants’ experiences of stigma and reported stigma practices, such as stigmatising behaviour and discriminatory attitudes; and third, the outcomes of these manifestations and wider health and social impacts, including social exclusion. The concept of stigma emerged from discussions about people’s understanding and perceptions about the disease.

### Drivers and facilitators of stigma

A lack of awareness about Parkinson’s in Kenya was perceived to be the main driver of people’s perceptions about the condition and its symptoms. Poor awareness in the general population was facilitated by the lack of health policy surrounding Parkinson’s in Kenya at the time, and the limited clinical capacity of neurological services, which made it difficult for people to obtain a diagnosis and make sense of their symptoms (see Fothergill-Misbah et al. 7 for challenges of diagnosis in Kenya).

“I’ve never heard anybody, [Parkinson’s has] never been featured in the newspapers, no doctors talk about it the way they talk about anything else. There is no awareness about it at all” (Angela, daughter of 78-year-old person with Parkinson’s)

All participants in this study had obtained a diagnosis and many did express their understanding of Parkinson’s as a medical condition. However, others also discussed alternative beliefs about the origin of the condition, for example: *“people think [the person with Parkinson’s] is bewitched or they think she is pretending or she’s drunk”*. Supernatural beliefs, including curses, witchcraft, or that someone was a “wizard” themselves, were common explanations for the “strange”, visible symptoms that people experienced. In contrast, several participants who self-reported a higher education level said they did not believe in witchcraft.

Supernatural beliefs were significant drivers of stigmatising perceptions, *usually* from people outside the immediate family (i.e., extended family, friends, church members or strangers), although on some occasions, it was participants’ spouses. Participants described how *other* people were more likely to believe in “sorcery”.

‘’There’s that whole thing of whatever you don’t understand is witchcraft...‘Some spirits are behind this whole thing’... There are some people who believe there’s no such thing as illness... So, there’s no way you’re going to convince such people that this person is just sick... The most meaningful [explanation] is witchcraft’’’ (Danny, son of 83-year-old person with Parkinson’s)

Evans-Pritchard [46] learned of similar reasonings for sickness and misfortune among the Azande in Central Africa – he wrote, *“surely these peculiar conditions demand an explanation”* (p.21), and the rational explanation was witchcraft.

*Why* someone had been cursed was a particular driver for stigmatising perceptions – often associated with blame. *Who* was responsible for the individual being cursed was placed on different groups: the person with Parkinson’s, family members, ancestors, friends, the devil (Satan), and God. One caregiver described how his mother’s church group convinced her that she was being “punished” for something she had done:

“Her church, they come and say, ‘This is your servant who has been faithful all these many years, why her?’ And so, they say, ‘Is it something you did?’… This idea was sort of put in her head by the rest of the church members” (Jasper, son of 78-year-old person with Parkinson’s)

The idea of punishment put the blame on the person with Parkinson’s, implying that they *“must have done something wrong”*, creating a form of internalised, or self-stigma. Alternative explanations included “jealousy” of the person with Parkinson’s and their successes in life.

In addition to the belief that curses could be placed directly on the person with Parkinson’s, several described the possibility of family curses (e.g., passed down from relatives).

“Worry, uncertainty, you know, what happened, what did we do wrong, what did she do wrong… We always have [curses] in mind, even after we knew it is Parkinson’s, there’s still that probably, maybe somebody, relatives, spoke, because we believe in those altars of witchcraft… There’s some people who can go out and curse you” (Yvonne, daughter of 77-year-old person with Parkinson’s)

Again, Evans-Pritchard identified similar beliefs among the Azande [46], where knowing the proximate cause of symptoms (e.g., Parkinson’s) did not preclude a supernatural ultimate explanation for a question that biomedicine could not answer. It also became clear that these beliefs about witchcraft were more common among participants with more pronounced, outward symptoms, which could be made worse by inappropriate treatment.

Closely linked with the idea of witchcraft was a fear of contagion from Parkinson’s, as one 58-year-old participant described, *“[people] think they could get sick like me”*, but also from the curse that was believed to have caused it.

“They’re like, ‘I don’t want to go to that house because whatever spirit is there might get into me’… There’s some people I don’t expect to ever see coming to visit because they believe that my dad is bewitched and if they come anywhere near him, the same thing might happen to them” (Danny, son of 83-year-old person with Parkinson’s)

Fear of contagion is a powerful driver of stigma that has also been observed in other ‘visible’, non-communicable neurological conditions, such as epilepsy, in Nigeria [47] and Cameroon [48].

Furthermore, participants reported how fluctuations in symptoms (dependent on ‘on’ and ‘off’ times) caused conflicting reports of the person’s condition, which could further contribute to the belief that supernatural forces were at play or that the person was pretending or malingering (also seen in Sweden; see Sunvisson and Ekman [49]), acting as additional drivers of stigma. Other drivers included prevalent stereotypes of Parkinson’s, particularly that it was a disease of “white”, “rich” or “old” people, facilitating negative societal attitudes towards those who did not fit this category. People with Parkinson’s reporting being labelled as “drunk” was also common, a stereotype also described from the USA (see Hermanns [33]).

Age was an intersecting stigma, with younger participants reporting more experiences of stigma than older individuals (described below). Some younger participants felt they were suffering from a disease of ‘older people’ when they should have been in their “youthful years”, being productive and “useful”. In contrast, Olivia described how she would take her 71-year-old father out in his wheelchair but said, *“[people] just think he is old”* and had respect for him because of his age. Older people were also expected to have some form of disability and deterioration as they aged, which meant that, even with Parkinson’s symptoms, this was largely perceived as “normal” old age. For a discussion on the expectations around ageing among participants, and how the progression of Parkinson’s fits within these expectations, see Fothergill-Misbah et al. 7.

### Stigma experiences and practices

Participants described their experiences (lived realities) of stigma, which included internalised (felt or self), anticipated and secondary (associative) stigma, as well as stigma practices (enacted stigma), which could reinforce prejudices towards people with Parkinson’s, fuelling social inequalities [21].

Participants reported multiple experiences of enacted stigma, that is, discrimination by others towards them because of their condition (stigma practices). This involved discrimination by strangers, wider family and friends, and, in some cases, immediate family members (e.g., spouses). Tuwile, a 58-year-old man with Parkinson’s, had very pronounced, outward physical symptoms, including dyskinesias (involuntary, erratic, writhing movements of the body) which could be shocking. He recalled people avoiding him on the street, taking a wide berth around him, crossing over the road when they saw him coming, vacating the seat if he sat next to them on the bus, and avoiding interaction with him. From Tuwile’s experience, and as discussed above, it became clear that participants who were younger and had more visible symptoms tended to experience, or report, more stigmatising perceptions than older people. Tuwile also made obvious efforts to hide his head-jerking movements, suggesting that the enacted stigma contributed to internalised or self-stigma.

Other participants acknowledged how families and friends had distanced themselves from the individual with Parkinson’s and their immediate family (secondary stigma), or *“disappeared into thin air”.*

“Most of [my friends] left me. You know, not many people want to associate with you when you have a strange condition… In fact, I had a list of about 100 friends, now I have about ten left” (Jacob, 33-year-old person with Parkinson’s)

Four younger male individuals also described instances of enacted stigma by their spouses, resulting partly from their inability to explain the origin of their symptoms, as well as their (reported) inability to continue working and providing for their families, which resulted in abandonment (see outcomes and impacts section). For example, Jacob described how his wife believed *“somebody had cursed [him] out of envy”*. He explained he had to acquiesce this belief for fear of losing her support.

“Initially I had to lie low when they [immediate family] came about with those ideas because I really wanted their support, you see, so I could seem like to agree with them. But I knew inside myself that it was not spiritual… I couldn’t be in a position where I could argue with my wife because what if she goes? That would be my worst… I’m afraid if that can happen” (Jacob, 33-year-old person with Parkinson’s)

Jacob was unable to work because of his condition, and here also describes a self-perceived dependency on his spouse, a form of self-stigma. Jacob also described feeling that he no longer ‘belonged’, adding: *“Sometimes, I feel out of place. Sometimes, I feel like I am a burden”.* Hermanns has referred to similar “invisible” manifestations of stigma in the USA, including concerns of being a burden [33]. Self-stigma was common and evident among participants who described feeling “sorry for themselves”, wanting to “give up”, or feeling that they were a “bother”. John, aged 65, described feeling *“hopeless”* because he could no longer communicate with people or work. He often excused himself from social situations, avoiding people and questions about his condition.

Caregivers also described situations where progressing symptoms resulted in withdrawal from social occasions. For example, Anya’s husband had difficulty with his mobility and speech as his condition progressed, which resulted in people not being able to understand him, and him being excluded from conversations in social situations. Occasions such as this could fuel internalised stigma and contribute to individuals with Parkinson’s having to come to terms with a changing ‘self’ as well as changing roles. These perceptions could be reinforced by others’ beliefs that people with Parkinson’s should not be working or participating in particular aspects of society, contributing to a form of internalised stigma about their ability. For example, Julian’s uncle with Parkinson’s sold flowers and plants on the side of a busy road:

“People used to ask why he is coming here [to work] or why we are letting him work. But we’d inform them that he has to exercise his hands and exercise himself… Everyone used to ask why we are letting him work, we should let him stay home” (Julian, nephew of deceased person with Parkinson’s)

In contrast, one participant described how her aunt had to continue working on her fruit and vegetable stall, otherwise people might think she was “pretending to be sick” so she could avoid work. Individuals had to constantly negotiate other people’s perceptions about their condition and ability.

Participants also described feelings of self-stigma that resulted from their embarrassment about their symptoms, for example, spilling food, needing a walking stick, or drooling (similar to findings in The Netherlands [35]). Similarly, caregivers reported instances of the person with Parkinson’s feeling embarrassed about having urinated in their bed, needing to use assistive devices, or requiring a toilet aide in their bedroom. Furthermore, perceived “clumsiness”, or fear of falling, which participants felt could be “embarrassing” contributed to them not leaving the house, spending more time indoors, and finding that their social life had “shrunk”. Participants with more discrete symptoms were able to “hide” their condition, yet still experienced self-stigma. As Mildred (aged 66) described, because her symptoms were not “obvious”, people were not quick to judge her appearance. She said she would feel “hurt” if she had visible symptoms because of what people might think about her, a form of anticipated stigma. Caregivers also described situations where the person with Parkinson’s would try to hide their symptoms, for example, holding their hands in their lap to stop their tremor.

### Outcomes and impacts

The different forms of stigma described had social outcomes, from isolation and abandonment to advocacy, and health outcomes, including barriers to seeking treatment, or use of alternative treatment. Subsequently, stigma resulted in wider health and social impacts, affecting quality of life, aggravating poverty due to loss of income, increasing the severity of disability and sometimes resulting in mortality.

Stigma experiences and practices contributed to people with Parkinson’s increasingly avoiding social situations, resulting in isolation and reduced social interactions. Several participants described feelings of loneliness as a result, and worsening mental health, perpetuating self-stigma experiences. Most participants were no longer able to engage in paid work, limiting their financial capacity and increasing their reliance on others for support. This also had consequences on their physical health, with reduced opportunities for mobility and exercise, an important aspect of the management of disease, and contributed to a reduced quality of life. Stereotypes of Parkinson’s (described above) could also delay treatment and health seeking, making their outward, visible symptoms more pronounced and noticeable as the disease progressed unmanaged.

In the case of 4 younger male participants, the enacted stigma they experienced resulted in abandonment by their immediate family. Tuwile’s wife and children had left him because he could not explain his condition or diagnosis; a consequence of challenges with awareness and clinical capacity, the resulting supernatural assumptions, and his inability to stay in employed work and support his family financially. He relied on donations from his church to buy sporadic medication and afford food, which had a detrimental effect on his health. Similarly, Magnus (55-year-old) described how he could not return home to his family in rural Kenya for 7 years because they associated his condition with *“witchcraft and superstitions”*, and he instead lived alone in the city.

In addition to the social outcomes that abandonment presented, the exclusion of individuals from their families also resulted in difficulties accessing timely and appropriate healthcare services and acquiring necessary medications, contributing to significant disease progression and deterioration in health and, in Tuwile’s case, death.

Supernatural beliefs could also result in the use of alternative therapies, for example, seeing a *“mganga”*, “witchdoctor” or *“special priest”* who could *“remove their curse”*. Participants described how friends and family suggested they should cease taking their biomedical medication and instead organise “*special prayers*” to *“cast out the demons”* causing their symptoms. These alternative therapies often resulted in worsening symptoms and could create tensions within the family. For example, Eunice, the spouse of a 79-year-old person with Parkinson’s, was accused by a family member of wanting her husband dead after refusing to give him ineffective and expensive herbal medicines. This demonstrates the pressure individuals and families were under to conform to expectations and certain behaviours, or risk further stigmatising perceptions and potential exclusion.

Despite the damaging health and social outcomes reported, there were some positives. Stigma can foster resilience and advocacy efforts among stigmatised groups – such as was seen with HIV [21]. Although not to the same scale as HIV and AIDS, participants in this study reported efforts to educate their communities that Parkinson’s was a *“real disease”* and raise awareness.

“I wish I could get a platform to make people know that there are people like that, there’s a condition like that, and they can get help and can live a normal life like normal people… so that they could not be jilted, they could not be like, cast out” (Jacob, 33-year-old person with Parkinson’s)

For some participants, their involvement in support groups was a way for them to surround themselves with people going through similar situations, validating the existence of ‘Parkinson’s disease’ as a biomedical diagnosis (the role of support groups in enabling legitimacy is explored by Fothergill-Misbah et al. 59. Groups were a safe space where attendees felt like they belonged and were not subjected to stigma.

## Discussion

This paper uses the Health Stigma and Discrimination Framework [21] and structural violence as an explanatory concept, to understand the experience of stigma related to Parkinson’s in Kenya. The framework serves as a tool to make sense of stigma as a process, highlighting key areas for intervention, and for comparison across diseases, disciplines, health issues and communities.

Identified drivers of stigma included a general lack of awareness about Parkinson’s, supernatural beliefs about the origin of symptoms and disease, blame associated with why someone was afflicted, fear of contagion from Parkinson’s (or the curse that may have caused it), beliefs about the person pretending or malingering, stereotypes (such as age), and fluctuations in symptoms, i.e., on and off times. The identification of these drivers highlights opportunities to intervene in the stigma process. However, it is possible that any efforts to tackle stigma are negated by the lack of policies and practices currently in place to support individuals.

Severe structural constraints facilitated the stigma process, notably, the lack of policy surrounding Parkinson’s and neurological disorders, the limited clinical capacity, poor accessibility of services and unaffordable medication, lack of financial protection from health costs, lack of social protection, and limited rights for people living with disabilities. These broader social and political processes that create widespread health and social inequalities, and create unequal distributions of power and resources, are what Galtung referred to as “structural violence” [25]. It is difficult to understand stigma around Parkinson’s in Kenya without exploring the role of structural violence in disadvantaging individuals [24], affecting the course of disease [26], and ultimately in facilitating the negative health and social impacts described. The study identified the dual challenge of a vulnerable group of people – those living with Parkinson’s – who were dependent on equally vulnerable people, with minimal state support.

This research has highlighted, in more detail than has been reported previously, the lived realities and outcomes of the stigma process for Parkinson’s. The lived experiences of stigma – including what literature refers to as self (or felt), enacted, anticipated and secondary stigma – impacted social relationships, impeded resource availability (social, material and financial), hindered access to health services and medication, contributed to stress and psychological responses, social isolation, and, ultimately, resulted in poor or sub-optimal health outcomes. Participants’ reports of being avoided on the street, excluded from social circles and communities, their inability to afford food or medication, and in some cases, being abandoned by spouses, significantly affected their quality of life, aggravated poverty, and increased the severity of disability, highlighting the significant public health issue of stigma. Those with lower social and financial capital appeared more likely to succumb to the consequences of stigma, as did younger individuals (intersecting stigma). These findings resonate with Goffman’s early work on stigma, spanning all three stigma “types”: physical, behavioural and social (or “tribal”) [17]. However, it is clear that the stigma process is more complex and dynamic than Goffman described, involving a process of labelling, stereotyping and separating, in a context of social, economic and political power [50].

These experiences from Kenya can be situated in the context of stigma across other communicable and non-communicable conditions. Health-related stigmas share commonalities across the socioecological spectrum and exploring these comparisons can contribute to our theoretical understanding of stigma as a process [21]. Having said that, stigma also uniquely affects lives in local contexts [51]. Understanding these unique social and cultural processes enables us to uncover the lived experience of stigma and develop refined and target ways to tackle it. This ethnographic research has shown the precise and nuanced ways in which stigma is experienced at a deeper level, and leads us to see stigma as a process, something that is embodied and enacted.

Parkinson’s is a visible disease, characterised by uncontrollable and involuntary body movements, i.e., tremor and dyskinesia, but also by “abnormal” behaviours as the disease progresses, e.g., hallucinations, apathy and cognitive impairment. These features of Parkinson’s have commonalities with other neurological conditions and, therefore, similarities in stigma practices and outcomes. For example, the very visible and unpredictable seizures characteristic of epilepsy, or the loss of inhibition or aggressive outbursts seen in dementia, have been reported to result in social rejection and exclusion in Africa, ultimately undermining the quality of life of individuals [52, 53]. A scoping review mapping evidence on stigma associated with non-communicable, neurological disease identified four key commonalities, including: attempts to conceal the condition, social exclusion, discrimination (and loss of power), and finally, lack of availability and accessibility of healthcare services [54]. The review reported higher levels of stigmatisation associated with the visible nature of neurological disease, highlighting the commonalities shared, and the opportunity to target stigma-reducing interventions across the spectrum of neurological conditions, which could have larger impact.

Literature on stigma relating to neurological disease (and wider disease areas), particularly from Africa, highlights the social exclusion individuals can experience, which is often associated with fear of disease manifestations, contagion and supernatural beliefs. These associations have also been acknowledged in Tanzania, Uganda and South Africa relating to Parkinson’s, suggesting that supernatural beliefs are likely to be widespread drivers of stigma on the continent, and have some of the most significant consequences for stigmatised groups. It is important to note that participants in this study were necessarily limited to those with the social and financial resources to access care in Kenya; those who have never accessed care may have unique experiences of stigma which require further exploration.

Hatzenbuehler, Phelan and Link write that stigma has a *“corrosive influence on health”* (p. 816) which manifests through the disruption of institutional and communal systems, interpersonal systems, and intrapsychic systems caused by the stigma ‘process’ [55]. They propose that stigma can be considered a *“fundamental cause of health inequalities”* (p. 819), affecting health outcomes, preventing access to resources which could otherwise minimise poor health, while perpetuating the reproduction of health inequalities among groups.

Having said that, stigma can also have positive outcomes [21], particularly relating to the formation of advocacy groups and efforts, as was seen with HIV and AIDS across the world. This was observed in Kenya through individual’s efforts to educate their communities, join support groups and improve the awareness of Parkinson’s. However, there is still a long way to go before Parkinson’s receives the same levels of advocacy and recognition as HIV and AIDS in Africa.

## Opportunities for interventions

Stigma has been described as a driver of morbidity and mortality at a population level [55], and a significant public health issue. Having uncovered the multiple stages involved in the stigma process and the deep and nuanced ways in which stigma affects individuals, it is imperative that interventions are put in place to tackle stigma and improve the lives of people living with Parkinson’s.

Cross et al. produced guidelines on interventions for stigma reduction, proposing that responses should change harmful stigma-related attitudes or actions, require multidimensional considerations [56] and include the following approaches: (1) training and contact, (2) rights-based strategies, and (3) a social capital strategy, i.e., use of social marketing. ‘Training and contact’ involves changing behaviours, improving knowledge and correcting false beliefs about the disease, including “myth-busting”, the promotion of empathy, and opportunities for discussion. ‘Rights-based strategies’ focus on support for affected persons, for example, removing barriers to access to healthcare and employment. Finally, social capital approaches can assist with the acceptability of a social idea or practice, in this case, Parkinson’s as the stigmatised condition through social marketing, for example [56].

Based on the three strategies above, findings from research in Kenya, and the goals of existing global Parkinson’s advocacy groups, I propose 7 activities for stigma reduction (expanded upon in Table 2), which are in line with one of the goals of the Intersectoral Global Action Plan on Epilepsy and Other Neurological Disorders 2022–2031 (IGAP) to “reduce the stigma, impact and burden of neurological disorders”. The activities under ‘rights-based strategy’, although beyond the scope of this paper, would address the structural violence and power imbalances that make reducing health disparities particularly challenging [55]. All proposed activities should be developed and implemented with input from people with lived experience and, where possible, support from existing advocacy and awareness groups. The success of education, training and public awareness campaigns will depend on effective co-design.

**Table 2:** Proposed activities and outcomes based on Cross et al. approaches to reduce stigma

Approach	Activity	Proposed outcomes
Training and contact	Educational campaigns for healthcare professionals (HCPs) and institutions	Increase awareness about Parkinson’s symptoms among HCPs, ensure timely and accurate diagnoses at primary care level & initiation of treatment
	Community health training and contact initiatives	Tackle myths and misconceptions & encourage earlier diagnoses through widespread awareness of symptoms
Social capital strategy	Establishment of support groups	Provision of support and resources & creation of a platform to advocate for rights
	Media and public education campaigns	Increase the profile and visibility of Parkinson’s & raise awareness to tackle stigmatising perceptions
Rights-based strategy	Increased provision of neurological services	Improve access to affordable, sustainable treatment and care for people with Parkinson’s
	Government subsidised medication	
	Access to health and social protection, and disability rights	

### Training and contact

Educational campaigns for healthcare professionals at the district, provincial and national referral hospital level [57] have the potential to reduce the negative attitudes towards individuals with Parkinson’s – particularly those relating to supernatural beliefs where a biomedical explanation is not available (see Fothergill-Misbah et al. 7) – and prevent the exclusion of people with Parkinson’s from society. At lower levels of public care in Kenya, community training and contact initiatives have the potential to tackle myths and misconceptions. These activities align with recommendations in the World Health Organization Technical Brief [58], which highlights primary care as the most logical setting to address Parkinson’s in regions where access to specialists is limited.

### Social capital strategy

The establishment of support groups would provide much-needed support to people with Parkinson’s and their families, educational resources, a safe space for individuals, and a platform from which to advocate for rights [59]. As a result of the Covid-19 pandemic and improved capacity to network globally, support groups can link with global organisations to facilitate advocacy efforts. Media and public education campaigns can also play an important role in raising the profile of diseases and creating awareness. Examples include successful public health messaging around diabetes (*sukari*) and high blood pressure (*presha*) in Kenya or anti-stigma campaigns for mental health (e.g., ‘Time to Change Global’, a social media anti-stigma initiative was piloted in Kenya in 2020 with positive results [60]). Increasing the visibility of Parkinson’s, and other related neurological conditions (e.g., dementia, epilepsy), will increase their profile as biomedical diseases, and reduce negative perceptions or associations with supernatural beliefs, creating more inclusive communities.

## Conclusion

This paper has shown, in more detail than previous studies [13–15], how people living in low- and middle-income countries such as Kenya experience stigma. Stigma has been described as a social determinant of population health [55] and this was evident from the significant consequences and outcomes of the stigma experiences and practices participants described. People with Parkinson’s in Kenya are already contending with insurmountable structural constraints and resulting “structural violence”. These constraints, including the limited accessibility and affordability of services and medication, and lack of health and social protection, facilitated the stigma process, made the enactment of agency over participants’ lives complex, hindered the optimal management of the condition, and significantly impacted participants’ health and wellbeing.

As the incidence and prevalence of Parkinson’s continue to increase globally, awareness of the disease and advocacy for its recognition in low- and middle-income countries needs to improve. One positive step towards achieving this is the release of the Technical Brief ‘Parkinson disease: A public health approach’ by the World Health Organization [58], which aims to promote mechanisms to strengthen countries’ capacities to respond to the public health challenge posed by Parkinson’s. Included in this brief is the need for advocacy and awareness-raising efforts to change public attitudes towards Parkinson’s, and recognition of the role support groups can play in improving population-level understanding of the condition. The brief, as well as the adoption of the Intersectoral Global Action Plan on Epilepsy and Other Neurological Disorders 2022–2031 (IGAP), have the potential to make a significant contribution towards strengthening services and support for neurological disorders, and ensure that brain health is a top priority for policy makers, particularly in low- and middle-income countries where it is most needed.

## Data Availability

The qualitative data generated and analysed during the current study are not publicly available as participants did not consent to data sharing and doing so could compromise anonymity and confidentiality. Data are available from the corresponding author on reasonable request.

## Footnotes

1 Patient advocacy groups have described their preference of the term ‘Parkinson’s’ over ‘Parkinson’s disease’, due to the reluctance to be labelled as having a ‘disease’. This paper refers to Parkinson’s throughout.
